# MitoLandscape, a semi-automated pipeline for subcellular localization and quantification of mitochondria

**DOI:** 10.3389/fcell.2025.1668779

**Published:** 2025-11-07

**Authors:** Enrico Negri, Virginia Fernández, Víctor Borrell

**Affiliations:** Instituto de Neurociencias, Consejo Superior de Investigaciones Científicas & Universidad Miguel Hernández, Sant Joan d’Alacant, Spain

**Keywords:** organelle, morphology, machine learning, computational biology, super-resolution, neurodevelopment

## Abstract

The precise characterization of mitochondrial morphology and subcellular localization provides crucial insights into cellular metabolic states and developmental fates. However, accurately analyzing mitochondria in cells with complex morphologies remains challenging, particularly within intact tissues where current methodologies lack sufficient resolution and specificity. Here we introduce MitoLandscape, an innovative pipeline tailored for comprehensive mitochondrial analysis at single-cell resolution in the developing nervous system. MitoLandscape integrates Airyscan super-resolution microscopy, semi-automated segmentation (leveraging ImageJ and 3DSlicer), machine-learning-driven pixel classification (ilastik), and a modular custom Python script for robust and versatile analysis. Using graph-based representations derived from manual annotations and binary mitochondrial images, MitoLandscape efficiently extracts detailed morphological parameters from distinct subcellular compartments, applicable from cells with simple morphologies to complex neuronal architectures. Additionally, the pipeline quantifies mitochondrial distribution relative to specific cellular landmarks, such as nucleus or soma. We validated MitoLandscape *in vitro* and in neural tissue, demonstrating its capability to precisely and reliably map mitochondrial features in diverse biological contexts. MitoLandscape represents a powerful, user-friendly, and highly adaptable solution for investigating mitochondrial biology in cell and developmental research.

## 1 Introduction

The morphology, size, and subcellular distribution of organelles play critical roles in defining the physiology, metabolism and even developmental fate of cells. Mitochondria are particularly important given their central function in energy metabolism, calcium signaling, apoptosis and cell differentiation processes (Namba et al., 2021; Tilokani et al., 2018; Friedman and Nunnari, 2014). While classically presented as elongated cylinders, mitochondria display a wide range of morphologies, from small spheres to simple tubes and tubular networks. Different mitochondria morphologies correlate with specific functional states, and affect the overall cellular response to physiological stimuli and pathological situations (Mishra and Chan, 2016). The subcellular localization of mitochondria is also key to the location of ATP production, reactive oxygen species production and signaling, and metabolic regulation (Schwarz, 2013; Sheng, 2014). Therefore, the detailed characterization of mitochondrial structure and distribution in any cell type of interest is key to understanding the fundamental aspects of its biology, development and disease.

The pressing need to determine the detailed morphology and localization of mitochondria, in an accurate and quantitative manner, clashes with the significant limitations of existing methods. Traditionally, mitochondrial analysis has relied essentially on manual or semi-manual methods, which are highly time-consuming, labor-intensive, and prone to inherent subjective biases and human error. For example, changes in the length of individual mitochondria in neural cells have been previously described performing manual analyses (Iwata et al., 2020), but applying such methods to large-scale studies, or to complex samples such as intact tissues, is not feasible. To overcome this critical limitation, recent advances in computational approaches using machine-learning-based image analysis technologies have enabled high-throughput analyses of mitochondria (Chu et al., 2022; Chaudhry et al., 2020). These computational methods offer significant improvements in efficiency and reproducibility but have been largely designed for cells that are either isolated and/or have simple morphologies, conditions rarely found in tissue. Some efforts have attempted to extend these approaches to three-dimensional tissue scenarios (Fogo et al., 2021), but limitations in image processing and analysis protocols render these insufficient for the unambiguous assignment of mitochondria to individual cells.

Existing computational methods for image-based analysis of mitochondria become particularly problematic in highly heterogeneous tissues that are structurally complex. The developing nervous system becomes particularly challenging when applying these established methods to intact tissue. In this context, cells often exhibit complex three-dimensional architectures, closely interwoven processes, and dense cellular arrangements, which dramatically limit the accurate segmentation and morphological analysis of individual elements. Neural progenitor cells, for example, are very densely packed and extend elaborate processes tightly intertwined with neighboring cells, making conventional segmentation techniques insufficient for accurately resolving individual cell structures and their associated mitochondrial content. Thus, there is a pressing need for advanced methods of analysis that can accurately quantify mitochondrial morphology and localization within intact tissues at single-cell resolution.

Here we introduce MitoLandscape, an advanced computational pipeline specifically designed for the accurate, robust and versatile analysis of mitochondrial morphology and their subcellular distribution within individual cells in the intact developing nervous system. MitoLandscape integrates Airyscan super-resolution microscopy with semi-automated segmentation approaches combining 3DSlicer software, machine learning-driven pixel classification via *ilastik* (Berg et al., 2019, 20) and customized Python scripts for detailed mitochondrial characterization. By employing a combination of manual annotations, computational segmentation, and graph-based analyses, our approach efficiently resolves mitochondrial morphologies and localizations within complex cellular architectures. We validated MitoLandscape using both cultured neural cells and fixed tissue samples, demonstrating its efficacy in accurately capturing detailed mitochondrial structural parameters, subcellular localization, and spatial relationships to key cellular landmarks. MitoLandscape can also be applied to study the architecture of complex branched structures such as neurons, by skeletonizing entire cells or cellular processes. This pipeline analyzes and measures the number and length of primary branches, and assesses structural characteristics of specific regions (i.e., the end-feet of Radial Glia Cells). Thus, our pipeline works across scales from individual organelles (e.g., mitochondria) to whole cells, allowing researchers to investigate cell morphology and cytoarchitecture in a semi-automated, quantitative manner. MitoLandscape provides an essential tool to investigate mitochondrial biology and cell structure at high-resolution within physiologically relevant contexts, significantly expanding the power of cell and developmental studies.

## 2 Methods

### 2.1 HEK culture

HEK293T cells were cultured following standard laboratory procedures. Briefly, cells were maintained in Dulbecco’s Modified Eagle’s Medium (DMEM) supplemented with 10% fetal bovine serum (FBS), 1% penicillin-streptomycin, and 1% L-glutamine, incubated under humidified conditions at 37 °C and 5% CO_2_. Cultures were passaged regularly to maintain approximately 70%–80% confluency.

For transient transfections aimed at sparse cell labeling, cells were plated on 24-well plates and transfected using GeneJet reagent (Thermo Fisher Scientific), following the manufacturer’s instructions. Specifically, transfection mixtures per well consisted of 1 µg of a floxed plasmid encoding membrane-targeted EGFP and mitochondrial-targeted dsRED (mito-dsRED), and 10 ng of a plasmid expressing Cre recombinase, resulting in sporadic recombination events and sparse cell labeling.

Cells were fixed 24 h post-transfection by directly adding an equal volume (500 µL) of fixation solution consisting of 8% paraformaldehyde (PFA) and 30% sucrose to 500 µL of the culture medium, achieving a final concentration of 4% PFA. Fixation was carried out for 5 min at room temperature, after which samples were rinsed thoroughly with phosphate-buffered saline (PBS) and stored appropriately for subsequent analyses.

### 2.2 Electroporation

*In utero* electroporation in mice was performed at embryonic day 12.5 (E12.5) targeting the neocortex. Pregnant females were deeply anesthetized using isoflurane, and the uterine horns were gently exposed. A total volume of 1 μL of DNA solution was delivered into the lateral telencephalic ventricle via pulled glass micropipettes, followed by application of five square-wave electric pulses (28–35 V, 50 ms duration, 950 ms interval) using a Cuy21EDIT pulse generator (Bex Co., LTD.) and round electrodes (CUY650P5, Nepa Gene). After electroporation, uterine horns were returned to the abdominal cavity, which was suture closed, and the female was returned to the home cage after full recovery from anesthesia.

*In ovo* electroporation in chick embryos was conducted at 4 days post-fertilization (dpf) as described elsewhere (Cárdenas and Borrell, 2021). Briefly, fertilized eggs were incubated at 38.5 °C until the desired developmental stage. On the day prior to electroporation, a small amount of yolk was aspirated to improve visibility. For electroporation, a window was open in the shell to access the embryo, and DNA was injected into the lateral telencephalic ventricle followed by the delivery of square-wave electric pulses (30 V, 5 ms duration, 5 pulses at 500 ms intervals) using a TSS20 Ovodyne Electroporator (MCI) and round electrodes (CUY650P3, Nepa Gene). Eggs were then sealed and returned to the incubator to continue development under standard conditions (38.5 °C). At the appropriate stage embryos were collected, fixed in ice-cold 4% paraformaldehyde (PFA), and brains were processed for immunohistochemistry.

Electroporation of perinatal ferrets was performed targeting the neocortex following a similar protocol as described previously (Borrell, 2010). Ferret kits aged postnatal day (P) 2 were anesthetized with isoflurane and placed in a stereotaxic system for intraventricular injection of ∼2 μL DNA solution. Electroporation was performed by application of five square-wave electric pulses (50 V, 50 ms duration, 950 ms interval) using a Cuy21EDIT pulse generator (Bex Co., LTD.) and round electrodes (CUY650P7, Nepa Gene). Following recovery from surgery, kits were euthanized, and their brains collected 1 day later.

DNA solutions used for both mouse, ferret and chick electroporations contained 1 μg/μL of CAG-mEGFP-T2A-mitoDsRed plasmid and 10 ng/μL of Cre-expressing plasmid.

### 2.3 Primary culture

Fresh neocortical tissue from electroporated mouse embryos was collected 24 h post-electroporation. Tissue processing was performed following previously described protocols (Wimmer et al., 2025), with minor modifications. Briefly, dissected cortices were rinsed in a base culture medium consisting of DMEM/F-12 supplemented with D-glucose (2.9 mg/mL), penicillin/streptomycin (5 U/mL), and amphotericin B (250 ng/mL). The medium was then replaced with Neurobasal medium supplemented with B27 without vitamin A, basic fibroblast growth factor (FGF, 20 ng/mL), and epidermal growth factor (EGF, 20 ng/mL). Tissue was mechanically dissociated into a single-cell suspension by gentle pipetting. The suspension was centrifuged at 1,200 rpm for 3 min, and the resulting cell pellet was resuspended in 1 mL of supplemented medium. Cell viability was assessed using trypan blue exclusion, and only preparations with a viability of 70%–90% were used for further culture. Cells were plated at a density of 2 × 10^6^ cells per well onto 6-well plates pre-coated with poly-D-lysine (0.1 mg/mL) and fibronectin (1.5%). Each well contained 2 mL of supplemented medium. Cultures were maintained at 37 °C in a humidified atmosphere with 5% CO_2_. The culture medium was refreshed 24 h after plating and every 2 days thereafter to eliminate floating cells and debris. Seven days after plating, cells were fixed with 4% paraformaldehyde (PFA) and subsequently processed for immunostaining against EGFP and DsRed.

### 2.4 Constructs

Floxed CAG m-EGFP-T2A-mitodsRed was produced by Vectorbuilder. pCAG-Cre, was a generous gift of M. Gotz (Pilz et al., 2013).

### 2.5 Immunohistochemistry

Ferrets were perfused transcardially with 4% PFA and post-fixed for 30 min at 4 °C. Brains were cryoprotected with 30% Sucrose and then were frozen and sectioned under a cryotome at 50 μm.

Chick embryonic brains were fixed with 4% PFA for 30 min, cryoprotected and frozen like ferret brains, and sectioned under a cryostat at 20 μm.

Brain sections and fixed primary cultures were permeabilized with PBS containing 0.25% Triton X-100, blocked in 10% of Normal Horse Serum and 2% Bovine Serum Albumin (BSA) during 2 h, followed by incubation with primary antibodies overnight in blocking solution, and then incubation with appropriate fluorophore-conjugated secondary antibodies. Primary antibodies used were: anti-GFP (1:1,000, chicken polyclonal, Aves Lab.) and anti-dsRed (1:1,000, Clontech). Secondary antibodies used were: Alexa555 anti-rabbit IgG (Invitrogen); Alexa488 anti-chicken IgY.

### 2.6 Imaging and deconvolution

To minimize acquisition-driven variability and maximize segmentation fidelity, 3D stacks were acquired using an inverted confocal microscope (Olympus FluoView FV1000) or an inverted super-resolution confocal microscope (Zeiss LSM 880-Airyscan Elyra PS.1) operating in Airyscan super-resolution mode (140 nm). Acquisition parameters (objective NA, refractive index, emission bandpasses) were selected to satisfy Nyquist sampling in XY and Z for each channel, lateral and axial resolving power follow the Abbe limits. Under our conditions, Airyscan processing yielded approximately 120 nm (XY)/350 nm (Z) at 488 nm and 140 nm (XY)/380–400 nm (Z) at 555 nm, improving signal-to-noise ratio and edge definition of mitochondrial structures and subcellular landmarks. Raw data were exported as “.czi” and deconvolved in Huygens Professional software (Scientific Volume Imaging) using the Deconvolution Express tool with default parameters. Deconvolved volumes were then submitted to the segmentation stage (e.g., ilastik classifier or Fiji Mitochondria Analyzer).

### 2.7 Image segmentation

Image segmentation was required to achieve the single cell resolution in the next analysis. Three-dimensional (3D) image stacks of the membrane signal channel were loaded into 3D Slicer (Figures 2A–E). In the Segmentation tool window, new labels are added (Figures 2B,C,E), one for each cell we intended to segment plus one for the background. We annotate individual cells trying to cover pixels with different levels of intensity within each cell. For the thinner processes, single lines along them were usually sufficient for proper segmentation. Particular care and additional annotations, such as the precise cell separation, were necessary along the boundaries between adjacent cells and processes. We used the background label in the empty space around the cells of interest to cover positive pixels belonging to cells or processes that were not being segmented. In case of cells with low intensity signal, it was necessary to better annotate the surrounding background areas to avoid label spilling into the negative pixels. The first and the last pictures of the stack were filled with the background label (Figures 2B,C, top and bottom) to obtain an output with the same dimensions of the original image stack. We used the *Grow from seeds* option and after the Initialization step, we refined the areas where the segmentation preview was not satisfactory, and we updated the segmentation. Additional rounds of refinement and updating were performed until the result was satisfactory (Figure 2F). The Segmentation preview was saved as the default “.seg.nrrd” format.

The output file was open in Fiji, where the MorphoLibJ package (Legland et al., 2016) was used to remove the largest label (corresponding to the background volume), and this set the background pixels to zero.

To segment the nucleus, we started from the DAPI channel and used a combination of median filtering and thresholding in Fiji.

For soma segmentation, we manually removed the processes from the segmentation outputs. For the following steps of the pipeline, it was important to have each nucleus or soma labeled with the same value of the corresponding cell. The output of nucleus thresholding was an 8-bit image with a positive pixel value of 255. Using the “Process > Calculator Plus > Operation: Divide” command where i1 and i2 were both the same binary picture (we left the default “k1: 1.0” and “k2: 0.0”), we obtained an 8-bit image where the positive pixel value was 1. We used again the same command with “Operation: Multiply” and “i1: result of the previous step” and “i2: cell segmentation image”. In the output, every nucleus value was the same as the label of cell segmentation.

To segment the ventricle, we combined manual tracing and thresholding from the membrane signal channel.

### 2.8 Distance maps

A Python function was written (indicated as *geodesic_dist* in the code) to calculate both distances from the nucleus (Figure 3A) and from the soma (Figure 3B). The function takes into account the shape of the cell and the processes and pixel resolution to calculate the distance appropriately. The inputs are two binary 3D numpy arrays with the same shape, one mask that determines the volume inside which to calculate the distance, the marker which is the origin point for the distance (distance inside is zero) and a 3D tuple with the pixel resolution. The function is based on the sckit-fmm library in Python (Pedregosa et al., 2011). The output is a masked array where the value of every pixel within the mask is set to the distance from the marker. In the case of the distance from the ventricle (Figure 3C), we opted to use Euclidean distance because there are no obstacles within the tissue to consider and it is computationally faster. For more complex tissue structure, the same geodesic distance function used for the distances of nuclei and somas may be used.

### 2.9 Cell compartment identification

A function was written to recognize processes as separate components attached to the soma (*cell_annotation* in the code) (Figures 4A–C). This function takes as input two binary 3D numpy arrays with the same shape: the cell and the soma masks (Figure 4A). It produces a numpy 3D array with the same shape where the background is filled with zeros, the soma is labeled with 1 and every process is labeled with a different value (Figure 4C). Minor processes shorter than a selected threshold are annotated as part of the soma (Figure 4D).

A function was developed (indicated as *apical_progenitor_compartment_annotation*) that automatically recognizes the processes belonging to the apical or basal side of the soma for each cell and annotate them accordingly (Figure 4E). The identity of the process is determined by the position of its starting portion (a basal process that bends towards the ventricle would still be properly recognized as basal). All these functions require the pixel resolution, which we obtained directly in Python using the aicsimageio library (Brown et al., 2021) to read the Tiff images.

Then, two functions were written to annotate cell types with branching structures (Figures 5A–C). They are based on the skeletonization of the cell and on the Strahler analysis of the branching structure (Strahler, 1952; Ledderose et al., 2014). Strahler numbers correspond to the distance from the extremities of the tree. A map is obtained where the most distal branches are annotated as 1, the branches immediately upstream as 2, and so on. This analysis requires a structure without closed loops. Structures with closed loops were solved by removing loops with *minimum_spanning_tree* function of the networkx package. We used the distance of each pixel from the soma as a weight for the trimming step, such that loops are preferentially removed away from the soma. This analysis requires root detection (the root end nodes without any annotation would be considered equivalent to any other end node). Since our pipeline already includes information about the soma position and the geodesic distance from the soma, it does not require an additional ROI containing the root; it assumes that the end node with the minimum distance to the soma is the root. The first function (*strahler_analysis*) needs cell and soma binary masks and pixel resolution and produces a network annotated with Strahler numbers, a unique id for each linear branch, and a dataframe containing the length of each component. If an annotated mask is provided, such as the output of the *cell_annotation* function, each branch will be additionally assigned to the respective compartment. The annotated output can be used as input of the second function *annotate_from_network*, which would create a new labeling of the cell binary mask. This step can be used for future analysis.

### 2.10 Mitochondria binarization

An ilastik (Berg et al., 2019) 2-class pixel classification model was trained (mitochondria pixel and background), using a few annotated images (Figure 6A) where mitochondrial signal spans a wide range of intensities as ground truth.

To segment isolated cells, such as ferret cortical neurons and Apical Radial Glia endfeet, we used a similar approach training the model to distinguish background from cytoplasmic and membrane signal.

### 2.11 Mitochondria morphology and localization analysis

Our *mito_analysis* function takes as input the binary mitochondrial image (which can be obtained with the preferred binarization algorithm) (Figure 6B) and the pixel resolution. A skeletonization step (Figure 6C) is performed and a graph object is generated. Each voxel is associated to a node and it is connected to all the neighbor nodes (in a 26-voxels neighborhood). Next, a standard morphological analysis extracts relevant information (length, volume, surface, number of branches, number of endpoints, number of junctions, branch diameter and sphericity). For each isolated mitochondrial element, a center was calculated as the graph component barycenter. The barycenter is the voxel that minimizes the distance from all the other nodes of the graph, taking into account the voxel resolution (Figures 6C,D). This information allows locating each element in its specific subcellular compartment. In case of extremely long mitochondria, which span multiple components, the final location assigned is the component where the mitochondria center is located. Mitochondrial volume was measured from the binarized mitochondria images as the number of positive voxels times voxel volume. The surface calculation algorithm was based on the script of MorphoLibJ (Legland et al., 2016). The diameter calculation algorithm was based on the Mitochondria Analyzer plugin (Chaudhry et al., 2020). The total volume of mitochondria was obtained for each component as the total number mitochondria voxels belonging to the specific component. The core mitochondria analysis (Figure 7A) function produces two outputs, a mitochondria features table and a graph objects from Networkx Python package that can be used for additional custom analysis.

The analysis was automated with the function *cell_analysis* (Figure 7B). It takes as input the binarized mitochondria, the annotated image, where each component is labeled with a different number (the output of *cell_annotation* (Figure 7C) or *apical_progenitor_compartment_annotation* (Figure 7D) for the process annotation, or *annotate_from_network* (Figure 7E) with the Strahler network for branching annotation), the distance map from the soma previously calculated, and the pixel resolution. Two tables are generated as output: the first contains the aggregate results for each labeled subcellular component (Supplementary Table S1) (label information stored in the “compartment” column; the second contains information for every mitochondrial element (Supplementary Table S2). The number of branches which was previously calculated is used in this step to classify each element as network, rod and punctus, following the work of Bakare et al. (2021). Additional functions were written to automate the analysis of multiple cells within the same image (function *picture_analysis* in the code), and other utility functions to plot and manage the different intermediate images generated in the pipeline. The full code is available on https://github.com/enricoenne/MitoLandscape. All mitochondrial binarization, skeletonization, and subsequent graph-based analyses were performed in 3D using the full Airyscan z-stacks.

To study the fine spatial distribution inside subcellular compartments, the function *process_analysis* was written to quantify the volume of mitochondria along the length and the thickness of the process (Figures 8A–D). This function could be useful to study the shape of processes even in the absence of mitochondria. The function to quantify process and mitochondrial volume along the process uses the distance from the soma (Supplementary Figure S1) to split the process in subsections of defined thickness (the default is 0.5 μm). Relying on the distance from the soma allows analyzing processes that are twisted; although subsections that are not perpendicular to the process axis cause a minor deformation, this usually does not alter the results considerably (Supplementary Figure S1). For the population analysis of apical Radial Glia Cells, we analyzed 102 cells (Figures 8I,J).

### 2.12 Datasets analysis and comparisons

The analysis of our HEK293T cell dataset was performed on 22 cells from 6 pictures. These cells show more complex 3D mitochondrial organization compared to apical Radial Glia Cells (Figure 9A). Cell, mitochondria and nuclear segmentation was performed as described previously.

The analysis of Cardiomyoblast cell-line H9c2 in glucose and galactose condition (Opstad et al., 2022) was performed on their FixedGA subset (10 pictures for galactose condition and 15 for glucose condition). The processed and aligned pictures were used (SIT_ALX). Both mitochondrial green channel (Figure 9F) and far-red lysosome channel (Figure 9F) were segmented with specifically trained 2-pixel classification models on ilastik. Models were trained on annotations on pictures coming from both conditions. Since pictures contained multiple partial cells with no way to distinguish one from the other or to obtain the full cellular volume, the pipeline analysis was performed on a picture scale, and the only comparable values are relative metrics, such as mean quantification of individual mitochondrial properties (Figures 9H,I), percentages (Figure 9J) and distributions (Figures 9K,L). Due to the heterogenous quality within the lysosome channel between the two conditions, it was not possible to perform reliable organelle analysis on the lysosome components, but the binarized output of the segmentation was used as a reference point for the process analysis function. This produced a distribution of mitochondrial density as a function of the distance from the lysosomes (Figure 9L). All the statistical tests and plot annotations are performed using Python library Statannotations (Charlier et al., 2022).

To test the reliability of our pipeline we compared its results on a HEK293T cell dataset of 8 pictures with Mitochondria Analyzer, an easy-to-use ImageJ plugin for morphological mitochondrial analysis. We used the standard settings both for local thresholding and for analysis, we chose “per-cell analysis”. Since the plugin does not allow for multiple cell identification within the same picture, we did not use any cell segmentation either for our pipeline, to obtain comparable outputs. The plugin is composed of two parts, a first local adaptive thresholding step followed by the morphological analysis. To account for the impact of thresholding/binarization step, which can be considerable (Hemel et al., 2021), we saved the output of the local thresholding and the output of our ilastik binarization model. We then performed both analyses, the plugin and our pipeline, on both binarized sets of pictures (Figure 10). To be able to compare the results we had to add a thresholding step based on the size of the element, because Mitochondria Analyzer automatically excludes particles below 0.05 µm^3^ of volume.

To observe the effects of different binarization steps to the downstream analysis we repeated the analysis of the HEK cell dataset (Supplementary Figure S2) using two different 2-class pixel classification ilastik models. The two models were purposely trained with a lower stringency annotation (Supplementary Figure S2B left) and higher stringency (Supplementary Figure S2B right).

## 3 Results

### 3.1 3DSlicer is a powerful tool for segmentation of complex cellular morphologies

**FIGURE 1:**
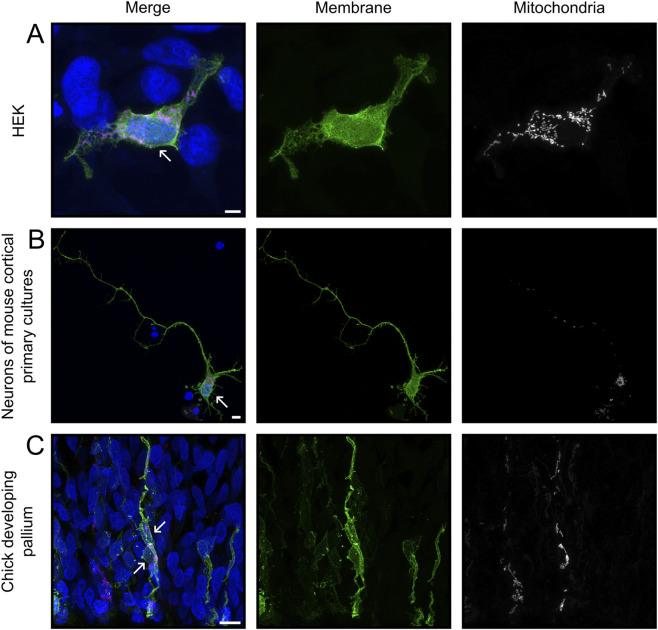
Input images for MitoLandscape. Representative maximum intensity projections of z-stack images from HEK293T cells **(A)**, neurons in culture **(B)** and Radial Glia Cells from chick embryo **(C)**, captured with Airyscan super-resolution and processed with Huygen’s deconvolution, illustrating different levels of sample complexity. Cells were transfected with plasmids encoding mEGFP (green) and mitoRed (magenta) to label cell membrane and mitochondria, respectively, and nuclei were labeled with DAPI. Arrows point at nuclei of cell of interest. Scale bars, 5 μm.

**FIGURE 2:**
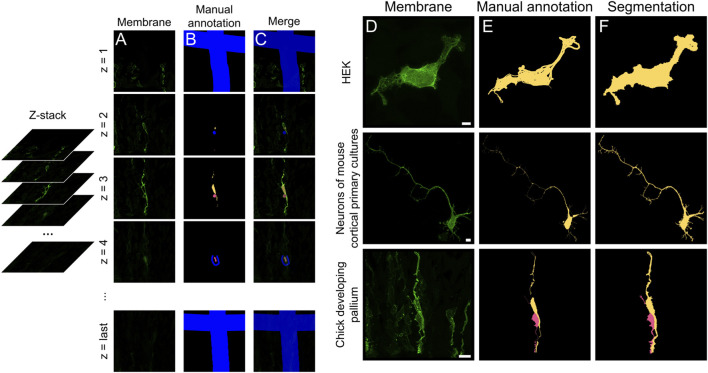
Semi-automated segmentation of complex cell morphologies using 3D Slicer. **(A)** Selected slices from the z-stack open in 3DSlicer, from first to last slice (z = 1 to z = N). **(B)** Partial manual annotation of individual cells and their processes performed on 2D planes. **(C)** Merge of annotation and membrane channels. Background is labeled in blue. To preserve the original z-stack dimensions, the first and last picture of the stack were partially annotated as background (**(B,C)**, top and bottom). **(D–F)** Maximum projections of membrane channel **(D)**, manual annotation **(E)** and final segmentation output **(F)**, referred to as “Segmentation preview” in 3DSlicer. Scale bars: 5 µm.

To test our semi-automated analysis tool, cells expressing membrane and mitochondrial reporter proteins were imaged (Figure 1). Cells labeled *in vivo* frequently overlapped, despite our strategy to obtain sparse labeling. In particular, neural progenitor cells exhibited extensively intertwined processes, making segmentation by standard methods very challenging (Figure 1C). To overcome this problem, we used the *Grow from Seed* segmentation tool available in 3DSlicer software (Fedorov et al., 2012). This allowed efficient segmentation by combining semi-automated segmentation of coarse objects, such as the soma of high-contrast cells, with manual annotations for complex and fine cellular processes. The *Grow from Seed* tool was reliable in segmenting membrane boundaries and texture, even in the most complex cases when manual delineation along the entire length of neuronal processes was necessary (Figures 2A–C; see Methods). Segmentation quality depended relatively on signal strength, but even cells with low signal intensities could be segmented with 3DSlicer if they were isolated and their edges easily recognizable (Figures 2D–F). Hence, 3DSlicer enabled highly detailed and accurate segmentation of complex cellular morphologies. The output was a standard label picture where every cell is associated to a different value. Fiji was used to assign value zero to background, necessary for the following steps.

### 3.2 Distance maps to extract sub-cellular compartment information

**FIGURE 3:**
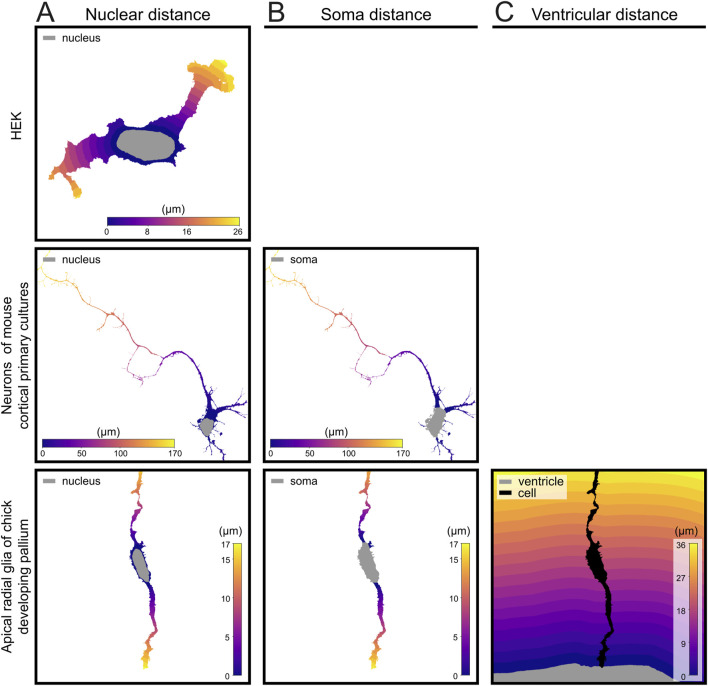
Distance map generation at increasing levels of biological complexity. **(A)** Geodesic distance from the nucleus in a HEK cell, mouse cortical progenitor cell and apical Radial Glia Cell from the developing chick pallium. **(B)** Distance from soma in mouse cortical progenitor cells and chick apical Radial Glia Cell. **(C)** Distance from ventricle of apical Radial Glia Cells from the developing chick pallium. Gray area in each distance map indicates the reference structure used to measure distance. Geodesic distance was used for nuclear and soma-based maps; Euclidean distance was used for ventricular distance calculations.

The specific localization of mitochondria is key for their function within cells, whether these are simple (Katajisto et al., 2015) or highly compartmentalized (Virga et al., 2024, 20). Even a very simple spatial analysis, such as measuring the distance of mitochondria with respect to the nucleus, already provides important information about their subcellular distribution. In the case of elongated processes such as neuronal axons or dendrites, for example, it is very informative to obtain the distribution of mitochondria along their extension. Accordingly, we wrote several functions to determine the length of individual cell processes, and the distribution along those of subcellular elements such as mitochondria. For simple cells like HEK, we wrote a function to simply measure distance from the nucleus (*geodesic_dist*), which can also be used in other, more complex cells (Figure 3A). For more complex cells containing thin processes that are well distinguished from the soma, like neurons and Radial Glia Cells, we used the same function to measure the distance from the soma along individual processes (Figure 3B). Finally, for cells attached or adjacent to a particular anatomical compartment, like apical Radial Glia Cells anchored to the ventricular surface of the embryonic telencephalon, we measured the distance along individual processes with respect to such reference landmark (i.e., ventricular distance; Figure 3C). Functions to measure distance along the cell relied on geodesic distance, thus allowing the algorithm to follow along the process no matter how twisted it was. Using the geodesic distance might introduce small errors when dealing with quantification of thick varicosities, which by definition are thicker than the main process, but we found that such deviations were negligible (Supplementary Figure S1). All these functions take as inputs the cell segmentation picture from the previous steps and the nuclear segmentation pictures (every nucleus is labeled with the value of the respective cell; see Methods Section 2.7).

**FIGURE 4:**
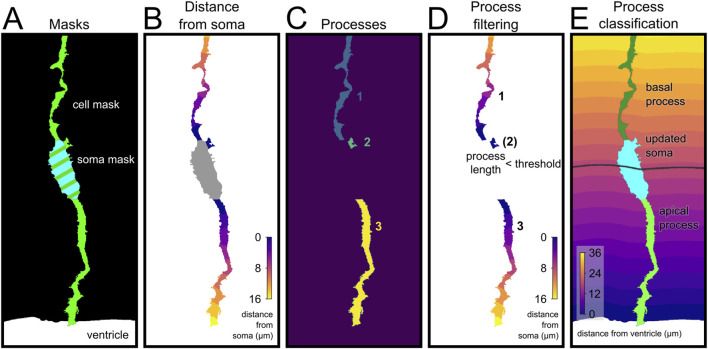
Automated annotation and classification of apical radial glia compartments. **(A)** Segmentation masks showing the cell (green), soma (cyan) and ventricle (white). **(B)** Geodesic distance from the soma (μm), used to define the length of each process. **(C)** Separate cellular components connected to the soma (each assigned to a numeric id). **(D)** The maximum distance from soma is used to calculate the length of the processes; short processes (<2 μm, number in bracket) were excluded from further analysis and reassigned to the soma. **(E)** Final classification of basal and apical processes. The short processes have been combined with the soma to obtain an updated soma. The soma distance from the ventricle (gray line) is used to classify the processes as apical or basal depending on the position of their attachment to the soma.

Finally, to analyze cells adjacent to an identified anatomical structure, we integrated in our pipeline a function to measure distance along individual processes with respect to the anatomical structure or border of reference (as a binary 3D picture). This is key when studying neural stem cells in the developing brain, like Radial Glia Cells that extend a process anchored to the ventricular surface or to a blood vessel (Ferent et al., 2020; Siqueira et al., 2018). As proof of principle, we analyzed the processes of Radial Glia Cells in the developing telencephalon, which display a very characteristic morphology extending an apical process away from the ventricular side of the soma, and a basal process away toward the opposite side (Figure 4; see Methods) (Viola et al., 2024). This analysis pipeline can be easily applied to measure distance along process with respect to other reference anatomical structures, such as blood vessels or amyloid plaques, as long as the feature of interest is contained within the segmented 3D image.

### 3.3 Analysis of neuronal and glial cell morphology

**FIGURE 5:**
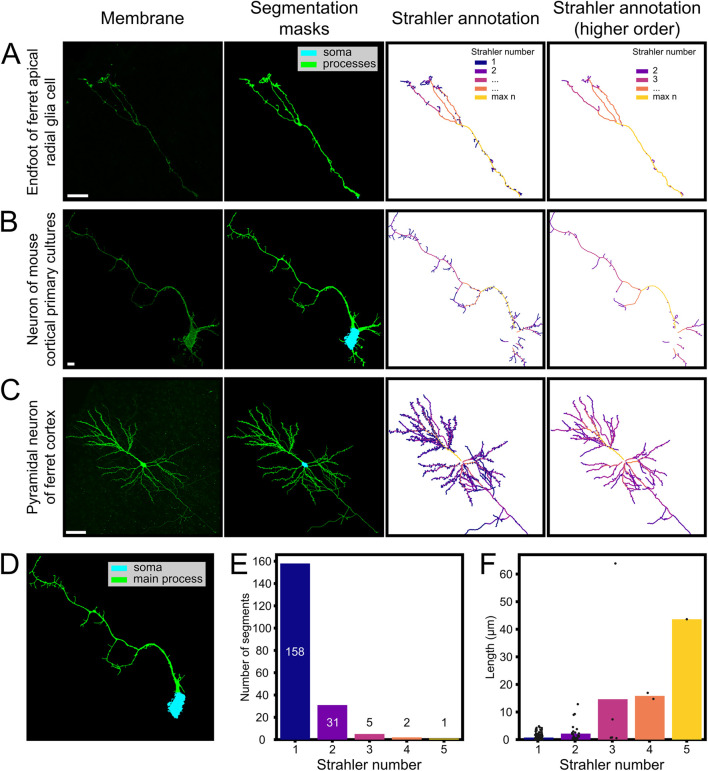
Automated skeletonization and branch analysis of neuronal and glial cells. Automated skeletonization and branch analysis of neurons and glia using the MitoLandscape pipeline. **(A–C)** Representative examples: **(A)** Ferret apical radial glia, **(B)** mouse cortical neuron *in vitro*, and **(C)** adult ferret pyramidal neuron (extracted from Borrell and Callaway (2002)); each showing membrane fluorescence, segmentation masks (soma/processes), and Strahler-order color-coded skeletons. **(D)** Soma and main process identification for compartmental analysis (mouse neuron from **(B)**). For ferret apical radial glia, the most proximal portion of the endfoot was annotated as root for the Strahler analysis. **(E,F)** Strahler analysis reveals abundant short terminal branches (order 1) and fewer, longer proximal segments of higher order. Scale bars, 10 μm **(A,B)**; 50 μm **(C)**.

To validate the broader applicability of our pipeline for cell morphology analysis, we applied it to analyze cells with branched structures, and annotated their detailed morphology. To this end we wrote additional functions (*strahler_analysis* and *annotate_from_network*) that we tested on fluorescent images from neurons and glial cells from a variety of preparations, containing neuronal dendritic trees and the terminal tree of Radial Glia Cell basal process (Figure 5). The first function (*strahler_analysis*) was an implementation of the Strahler analysis (Strahler, 1952). This annotates branches depending on their distance from an end point and outputs the associated network, with each node representing a pixel of the skeletonized processes (Figure 5). A second function (*annotate_from_network*) uses the Strahler numbers calculated previously to annotate the binary cell mask. In each case, MitoLandscape’s segmentation and skeletonization modules were used to extract the morphological structure of the cell of interest, converting the cell’s fluorescence signal into a mathematical skeleton representation. We tested three distinct types of samples: a) the branched end-foot of a Radial Glia Cell in postnatal ferret brain; b) cultured cortical neurons from mouse; and c) pyramidal neurons in the adult ferret cerebral cortex. These different types of samples represented a broad spectrum of cell sizes, complexities and imaging conditions (Figures 5A–C).

In the developing cerebral cortex, Radial Glia Cells extend a long basal process that arborizes profusely within the marginal zone just before contacting the basal lamina. Using MitoLandscape we isolated the basal process of a GFP-labeled Radial Glial Cell from the surrounding tissue (Figure 5A). The segmentation accurately captured the thin radial fiber together with its broader and complex terminal arbor. After skeletonization, the branched structure was clearly delineated, and the pipeline detected several branch points, effectively mapping its complexity (Figure 5A, right panels). From this skeleton, we obtained the number of branch segments and the length of each branch. These features are of great interest because the branching pattern of Radial Glia end-feet reflects how these cells interact with the surrounding environment and the basal lamina.

Next, we extended the analysis to neurons, which have an even more complex morphology than Radial Glial cells. We used neurons grown *in vitro* and developed *in vivo* (Figures 5B,C). MitoLandscape successfully segmented the cell soma and dendritic processes from the fluorescent membrane marker and produced a clean skeleton of dendritic branches. The skeletonization preserved all major dendrites and axonal processes, reducing them to their centerlines (Figures 5B,C, right panels). Branch endpoints were clearly identified in the skeleton’s graph representation. Depending on the needs of the study, the terminal branches of dendrites, i.e., dendritic spines, may be either excluded from the analysis to focus on primary dendritic shafts, or maintained to also analyze their quantity, distribution and length (Figure 5C). MitoLandscape also supports compartment-specific analyses. Using the segmentation mask, the soma (cyan) and main processes (green) were clearly identified (Figure 5D). This segmentation allowed the morphological quantification within distinct cellular compartments. For example, MitoLandscape automatically counted the number of branches in individual dendrites and measured their length (Figures 5E,F).

The reliability of these analyses depended again on the quality of the original images and the fidelity of cell segmentation. If two branches were separated by a distance smaller than the spatial resolution of the segmented image, annotation functions were prone to error in branch identification. Similarly, *strahler_analysis* could not deal with structures containing loops. To solve this, we extracted an acyclic graph favoring the opening of apparent cellular loops at the side most distal from the soma. Alternative methods to solve loops, such as removing pixels with the lowest intensity, could not be applied because our analyses were based on segmented binary pictures.

Altogether, our results demonstrated that MitoLandscape can quantify in a fully automated manner classic morphological features of neurons, such as the number and length of dendritic branches, as well as overall dendritic length. These features are traditionally measured in studies of neuronal development and plasticity using manual tracing or specialized software; MitoLandscape extracts them directly from images with minimal user intervention.

### 3.4 Mitochondria analysis pipeline

**FIGURE 6:**
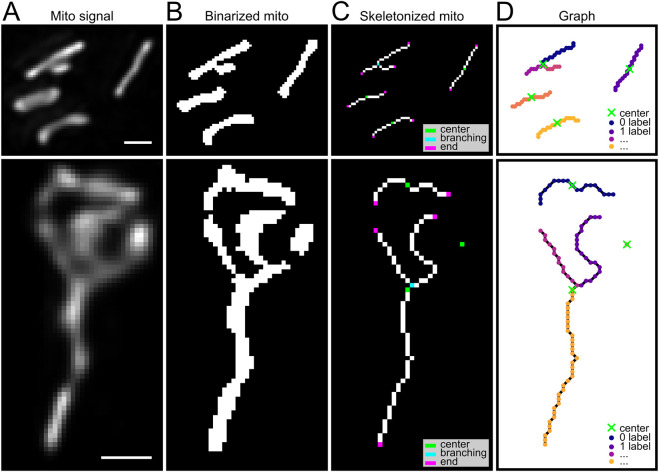
Mitochondrial skeletonization and graph-based analysis, example from chick developing pallium. **(A)** Raw Airyscan super-resolution microscopy images were deconvolved using Huygens software (Mito signal), maximum projection. **(B)** Maximum projection of binarized mitochondria was generated by a two-class pixel classification model in ilastik (Binarized mito). **(C)** Skeletonized representation showing endpoints (1 neighbor; magenta), branching points (more than 2 neighbors; cyan) and centers (green) for each mitochondrial element, calculated as barycenter of each mitochondrial element, treated as a graph where every voxel is a node connected to the neighbors. **(D)** Graph representation of mitochondria, green crosses indicate element centers, colors represent the single linear components (or branches). Scale bar, 0.5 μm.

Next, we went on to establish an analysis pipeline for mitochondria morphology and subcellular distribution. Our analysis started with the binarization of the image channel reporting the mitochondrial marker. Since mitochondria had different levels of intensity depending on the amount of reporter plasmid expressed, we found that thresholding methods were unreliable for binarization. Previous analysis pipelines delt with this issue performing the analysis with a range of threshold values (Zamponi et al., 2018, 201), or based on videomicroscopy images where the quality of a threshold could be evaluated based on the consistency of its output along consecutive frames (Lefebvre et al., 2021). In order to design a pipeline suitable for images on fixed tissue, where a stable level of intensity cannot be guaranteed, we used ilastik to obtain a 2-pixel classification model (Figures 6A,B). To ensure reliable results across any sample, we trained this machine learning-based resource with cells displaying a wide range of mitochondria signal intensities. Our analyses were conducted in 3D, ensuring that mitochondrial morphology and connectivity were quantified across the entire z-stack rather than from 2D projections. Nevertheless, mitochondria analysis was always limited by the resolution of original images and the local density of mitochondria. It was not possible to distinguish two mitochondria positioned closer than the resolution limits of Airyscan super-resolution microscopy (approximately ∼120 nm in the XY plane, ∼350 nm in Z), nor to fully resolve the fine ultrastructural details of a single mitochondrion with highly intricate morphology. Importantly, our pipeline includes the possibility of loading images already binarized to continue with the following steps, as both *mito_analysis* and *cell_analysis* function use as input a simple binarized 3D picture. In this way the user is free to employ any binarization method of preference, as tested with the adaptive thresholding output of the Mitochondria Analyzer ImageJ plugin.

After obtaining binarized images, we skeletonized the binary signal to extract the fundamental morphological structure of individual elements (mitochondria - total length and number of branches; Figure 6C) and to classify them (Bakare et al., 2021). To assign each element to a subcellular localization, we defined a center point based on the barycenter of its graph representation (Figure 6D). The center is chosen as the voxel with the minimum distance from all the others in the graph, as a way to keep into consideration the complexity of its branching structure. This enabled us to assign each element to the specific compartment to which it belonged. We set the analysis pipeline so that each element can only belong to one compartment, although this may be inaccurate in special cases, for example, with very long mitochondria that may extend along different subcellular compartments.

**FIGURE 7:**
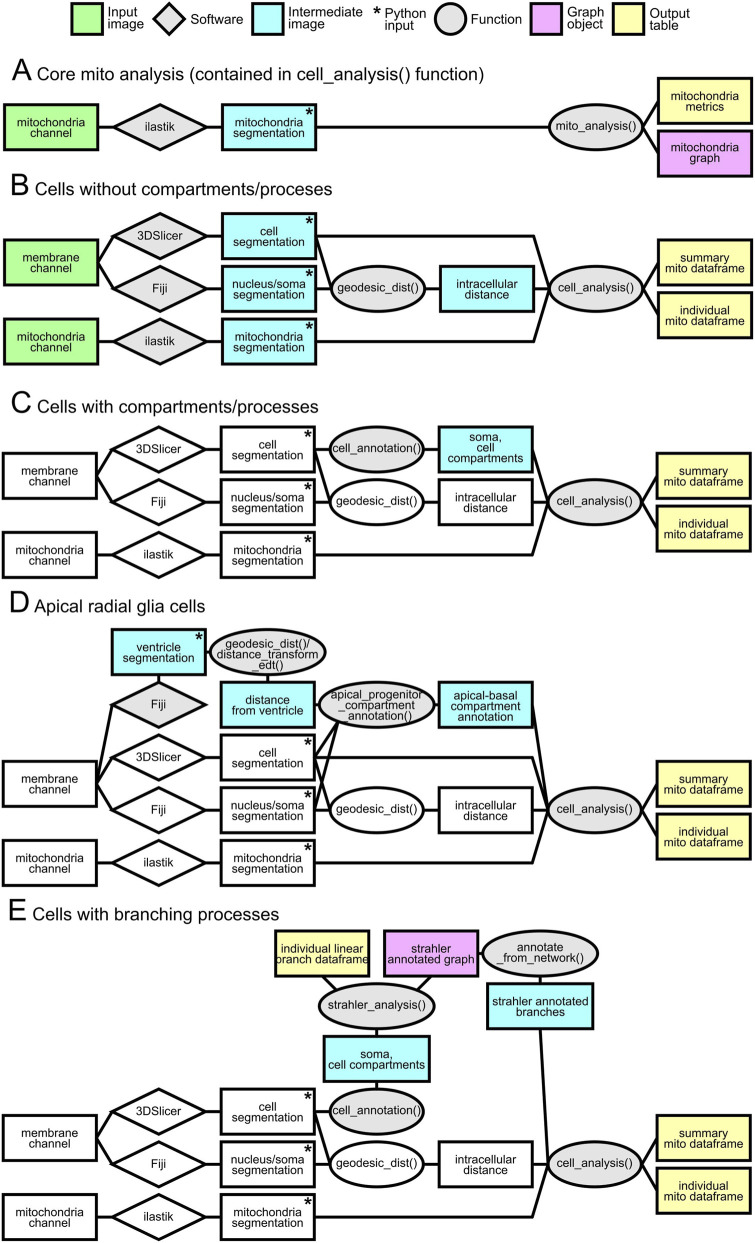
Schematic of analysis pipelines. Rectangles denote images or data products: green, input images (e.g., deconvolved acquisitions); blue, intermediate images required by the pipeline; pink, graph objects (NetworkX); yellow, output tables. Star marks the images used as input to the Python scripts. Steps invariant relative to the baseline pipeline (cells without compartment/process annotations) are shown in white. Gray rhombuses indicate third-party software used for manual or semi-automated segmentation; gray ellipses indicate in-house Python functions used within the pipeline. Panels: **(A)** core mitochondrial analysis—segmentation-derived, graph-based quantification; **(B)** basic cell analysis using nucleus/soma distance only; **(C)** analysis of cells with soma-derived compartments or other compartment annotations; **(D)** dedicated pipeline for apical radial glia exhibiting apico–basal polarity; **(E)** analysis of branching morphologies and associated compartments.

The overall analysis strategy, summarized in Figure 7, relied on modular pipelines tailored to specific data types and morphological complexity. Input images (e.g., deconvolved acquisitions) were processed through standardized preprocessing and automated or semi-automated segmentation by third party software (Fiji, ilastik, 3DSlicer), followed by custom written Python functions. The core pipeline quantified mitochondrial features from segmentation using a graph-based representation (Figure 7A). For simpler geometries, a basic workflow estimated subcellular position from the nucleus–soma distance (Figure 7B). For cells with explicit compartment annotations, analyses were extended to compartment-resolved quantification (Figure 7C). A dedicated workflow accommodated apical Radial Glia to account for their apico–basal polarity (Figure 7D). Finally, branching morphologies and their associated compartments were handled by a specialized pipeline (Figure 7E). Across pipelines, intermediate products included graph objects and, ultimately, standardized data tables for downstream statistical analyses, providing a systematic and reproducible readout of cellular features.

### 3.5 Analysis of mitochondria morphology and localization

**FIGURE 8:**
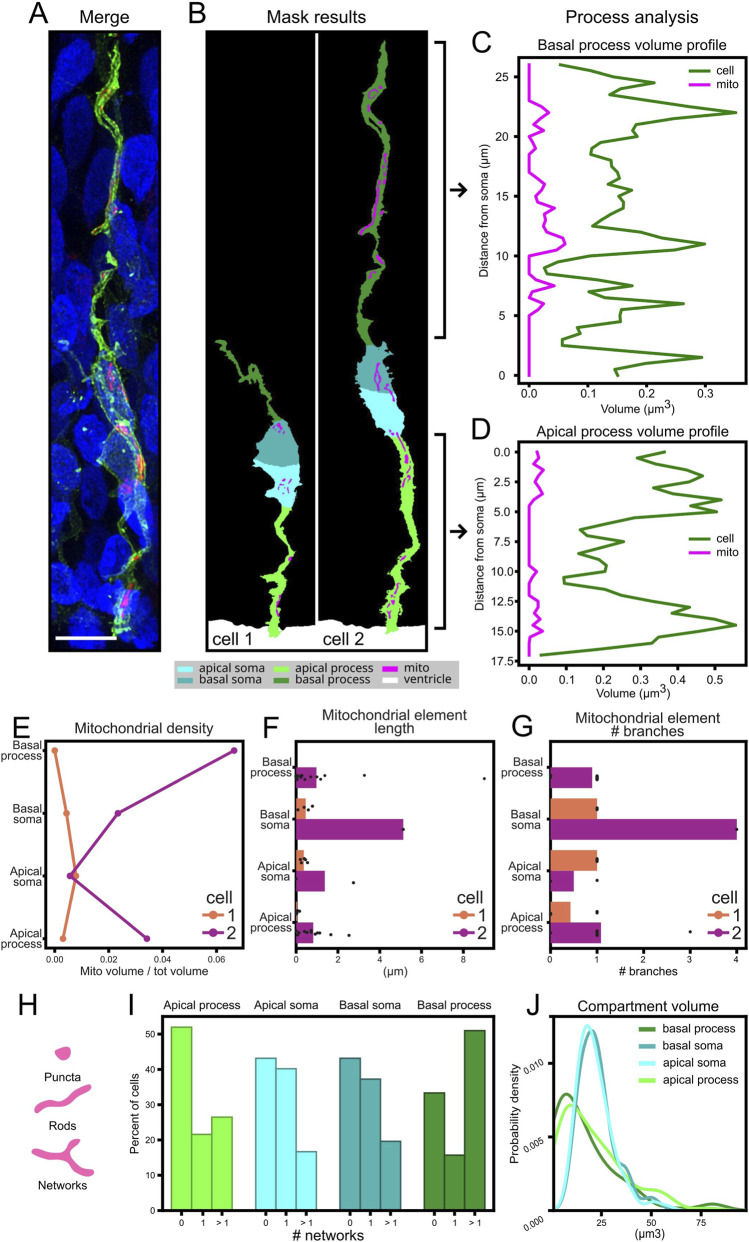
Comprehensive analysis of mitochondrial morphology and distribution. **(A)** Merged image showing membrane (green), mitochondria (magenta), and nuclei (blue) in an apical radial glia cell from chick developing pallium. **(B)** Segmentation masks identifying cell compartments in two cells of interest: apical/basal part of the somas and processes (cyan, magenta, light and dark green), mitochondria (magenta), and ventricle (white). **(C,D)** Volume profiles along the basal **(C)** and apical **(D)** processes, showing mitochondrial (magenta) and process (green) volumes as a function of distance from the soma. **(E)** Results of the analysis of the mitochondrial density across cellular components in the two cells of interest. **(F)** Result of the analysis of individual mitochondrial length and their subcellular distribution in the two cells of interest. **(G)** Result of the analysis of individual mitochondrial number of branches and their subcellular distribution in the two cells analyzed. **(H)** Morphological classification of mitochondrial element (Bakare et al., 2021). **(I)** Result of the network content of subcellular compartments in 102 apical radial glia cells from the developing pallium. **(J)** Kernel density estimation (KDE) plot of subcellular compartment volumes. Scale bar, 5 μm.

For a proof-of-concept and quality testing of our analysis pipeline, we focused on the analysis of apical Radial Glia Cells. In general, mitochondria are not single dots but three-dimensional elements with measurable volume, and their distribution along cells may be quite heterogeneous (Figures 8A,B) (Kuznetsov and Margreiter, 2009). Using MitoLandscape we quantified this heterogeneity by assessing the mitochondrial volume in each cellular compartment (as an aggregate) and the profile of mitochondrial volume along the apical and basal processes (Figures 8C,D). To normalize for potential changes in volume along cells and processes, we computed variations in mitochondrial volume alongside with cytoplasmic volume. The analysis outputs captured multiple mitochondrial features of each individual cell, from more global features like density and length (Figures 8E,F) to more detailed aspects like their branching (Figure 8G). MitoLandscape allowed this analysis in the different subcellular compartments at the level of individual cells, and as a population distribution. For example, the abundance of mitochondria with network structure (Figure 8I), or cell morphological features like the volume of subcellular compartments (Figure 8J).

**FIGURE 9:**
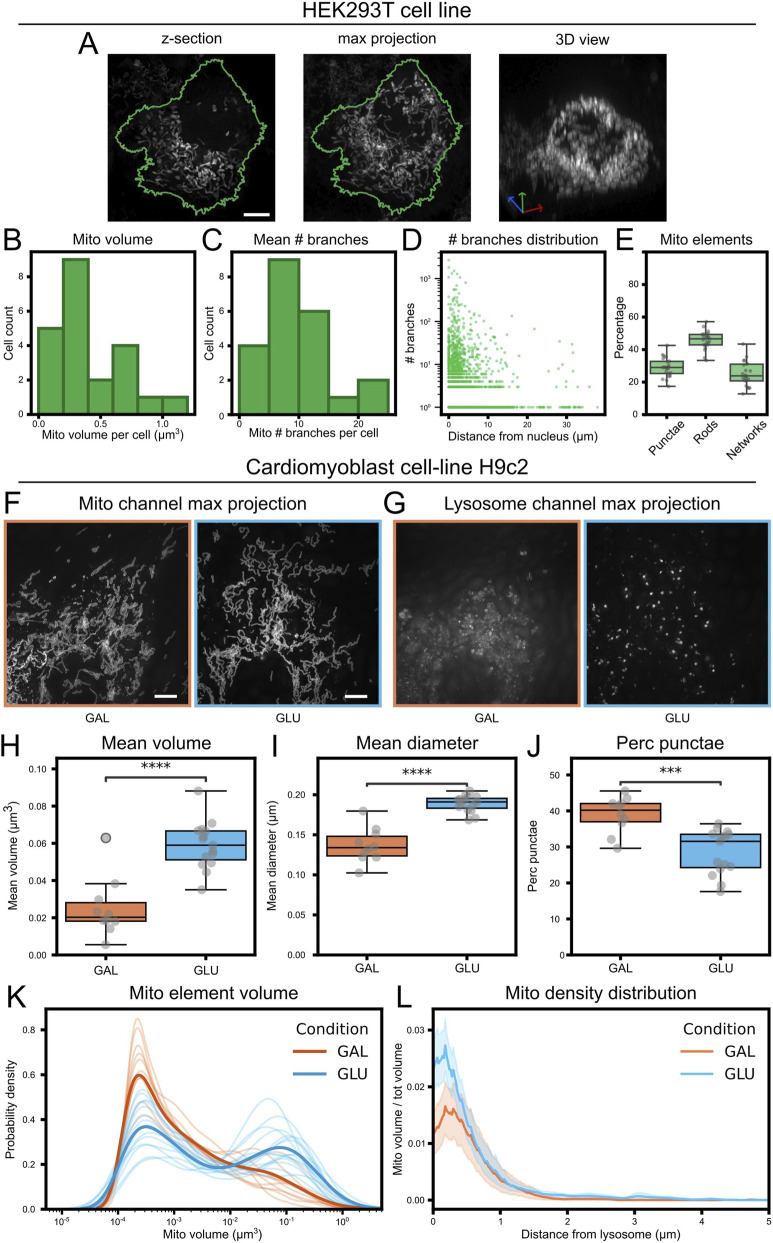
Cell culture datasets analysis. **(A)** Representative HEK293T cell: left, z-slice; middle, max projection; right, 3D rendering of the mitochondrial channel. In green the cellular outline from the max projection. **(B)** Histogram of the mean mitochondrial element volume per cell. **(C)** Histogram of the mean number of branches of mitochondrial elements per cell. **(D)** Branch number of individual mitochondrial elements as a function of their distance to the nucleus. **(E)** Population fractions of mitochondria morphological classes: punctae (spherical, unbranched), rods (linear, unbranched) and networks (branched). **(F)** Max projection of the mitochondria channel in H9c2 Cardiomyoblasts cultured in galactose-based medium (GAL; n = 10 images, orange) versus glucose containing medium (GLU; n = 15 images, light blue). **(G)** Max projection of lysosome channel. **(H–J)** Between-condition comparisons: **(H)** mean mitochondrial element volume per image, **(I)** mean mitochondrial diameter per image; **(J)** percentage of punctae per cell. **(K)** Kernel density estimation (KDE) plot of individual mitochondrial element volumes; thin lines, individual pictures; thick lines, average; colors represent condition (orange for GAL, light blue for GLU). **(L)** Mitochondrial density plot as a function of the distance from lysosomes; thick lines represent mean distribution; colors represent condition (orange for GAL, light blue for GLU). T-test. ***: p-value <0.001, ****: p-value <0.0001. Scale bar, 5 μm.

To evaluate the robustness of the analysis pipeline, we used a dataset of HEK293T cells (Figure 9A). The analysis of the distribution of relevant mitochondrial features, such as volume, branching and morphological complexity showed highly reproducible results across cells (Figures 9B–E), demonstrating the robustness of MitoLandscape. In this reductionistic biological context, MitoLandscape could also analyze the subcellular spatial distribution of mitochondria, using the position of the nucleus as reference (Figure 9D).

We then tested the sensitivity of MitoLandscape to detect differences in mitochondria profiling. To this end we analyzed a mitochondrial dataset of Cardiomyoblast cell-line H9c2 (Opstad et al., 2022) where cells were subject to different nutritional regimes, thus affecting mitochondria biology (Figures 9F,G). MitoLandscape was able to robustly identify significant differences between cells treated with standard glucose medium, or glucose-deprived galactose medium, in mitochondria volume, diameter and structure (Figures 9H–K). Similar to the analysis of distribution with respect to the cell nucleus, MitoLandscape also provided information on the spatial distribution of mitochondria with respect to other subcellular elements, such as lysosomes (Figure 9L).

### 3.6 Comparison of MitoLandscape to other analysis pipelines

**FIGURE 10:**
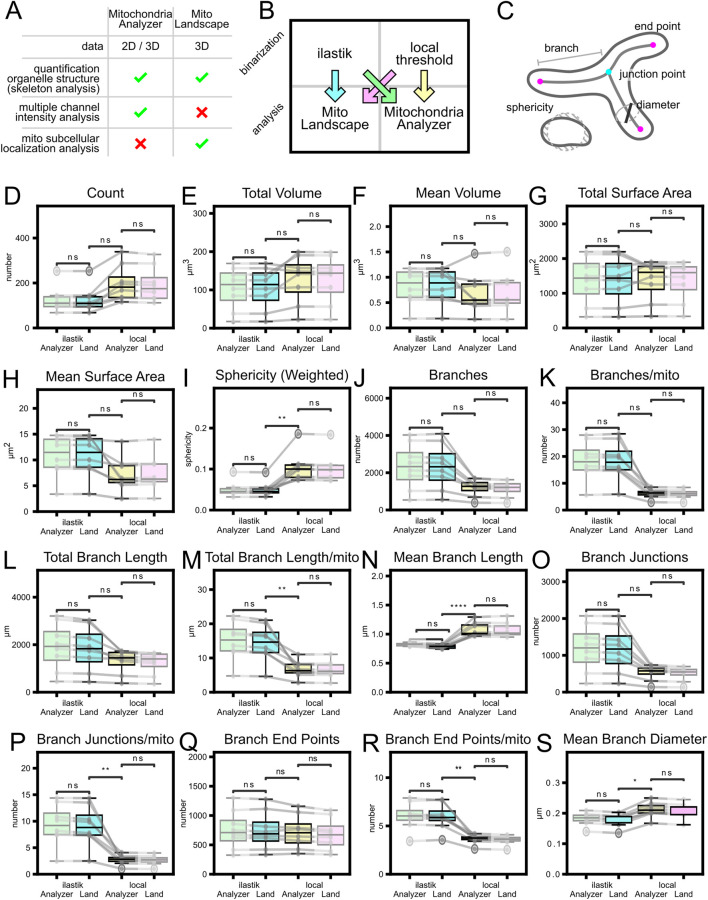
Comparison of analysis pipelines and the dominant effect of binarization. **(A)** Main features comparison between MitoLandscape and ImageJ plugin Mitochondria Analyzer. **(B)** HEK cells comparison (8 pictures) was performed following four combinations of parameters: blue, MitoLandscape on ilastik masks; yellow, Mitochondria Analyzer on its own masks; green, Mitochondria Analyzer on ilastik masks; pink, MitoLandscape on Mitochondria Analyzer masks. **(C)** Mitochondrial morphological features measured by the two pipelines. **(D–S)** Comparison of metrics distribution across the four different combinations. Gray lines connect same pictures. T-test with Bonferroni correction. *: p-value <0.05, **: p-values <0.01, ***: p-value <0.001, ****: p-value <0.0001.

When compared to other mitochondria analysis pipelines, such as Mitochondria Analyzer (Chaudhry et al., 2020) our pipeline shows comparable results (Figure 10). Our analysis produces the same types of metrics about mitochondrial number, size and complexity. Differences on the results between the two pipelines related essentially to the binarization step, as starting from the same mitochondrial segmentation files led to similar outcomes (Figures 10A,B; blue and green comparisons are as similar as pink and yellow). The effect of the binarization was also considered comparing the results of the HEK dataset using different ilastik mitochondria binarization models (Supplementary Figure S2). This showed that, as expected, mitochondrial volume and branch length were directly correlated with the stringency of the binarization model. More interestingly, both in the total number of mitochondrial elements and total number of branches we observed a trend where the number decreased for both higher and lower stringency. The former case had many false-negative pixels, whereas in the latter case the abundance of false-positives caused the merging of separate elements. This trend was already described in the literature when analyzing mitochondria at different background thresholds (Zamponi et al., 2018).

## 4 Discussion

Here we present MitoLandscape, a flexible and semi-automated analysis pipeline aimed at analyzing the morphology and spatial distribution of mitochondria at single cell resolution, particularly useful in complex tissue architectures. The motivation behind this development was to address a well-known technical limitation in subcellular biology: the difficulty of extracting organelle-specific data from individual cells within densely packed and morphologically complex tissues, where most conventional segmentation and analysis methods fall short in resolution, scalability and/or specificity. By combining super-resolution Airyscan imaging with machine-learning segmentation and a custom graph-based analysis in Python, our semi-automatic tool performs a detailed study of mitochondrial architecture and subcellular distribution, even in cells with long, branched, and/or intertwined processes.

One of the main strengths of our approach is its ability to assign mitochondrial structures to specific cells reliably, even in cases where processes are overlapping or in close proximity. This represents an important improvement over previous studies that depend heavily on manual annotation. For example, Iwata et al. (2020) provided high-quality mitochondrial quantification in neuronal cells through manual tracing, which while precise, is not feasible for large datasets or intact tissues. In tissue-based analyses (e.g., (Baumann et al., 2025, 2; Fogo et al., 2021)), authors were unable to assign mitochondria to individual cells due to high tissue density. Similarly, in several *in vitro* studies (Helguera et al., 2013; Meshrkey et al., 2021; Xu et al., 2022) mitochondrial morphology was analyzed at the population level, without single-cell resolution. In such contexts, our pipeline offers major advantages by enabling precise cell-specific mitochondrial quantification, improving interpretability and biological insight, while preserving spatial context and reducing user-dependent variability. Moreover, our approach complements previous 3D analyses of mitochondrial networks (Mitra et al., 2009) by assigning organelles to individual cells and linking them to specific subcellular compartments and tissue landmarks within intact tissue. Beyond earlier approaches, several recent frameworks (MitoSkel (Zaghbani et al., 2025), MoDL (Ding et al., 2025), Pycytominer (Serrano et al., 2025) and Nellie (Lefebvre et al., 2025)) advance deep-learning segmentation, feature extraction and standardized profiling. In contrast, MitoLandscape focuses on cell- and compartment-resolved 3D spatial quantification in intact tissues, with explicit mitochondria-to-cell/landmark assignment and geodesic, graph-based metrics that preserve anatomical context.

To validate the robustness of our approach, we compared the MitoLandscape analysis pipeline with a widely used method, the Mitochondria Analyzer Fiji plugin (Chaudhry et al., 2020). We performed this comparison using HEK cells, a standard model for *in vitro* studies. As detailed in Figure 10, we processed the same deconvolved images of the mitochondrial channel using both pipelines. Specifically, we used our custom ilastik model for binarization to feed the MitoLandscape analysis (Figure 10, blue) and the local threshold to feed Mitochondria Analyzer plugin (Figure 10, yellow). To further evaluate the impact of the binarization step, we also initiated both analyses with the binarization output from the other method: MitoLandscape on the local thresholding output of Mitochondria Analyzer (Figure 10, pink), and Mitochondria Analyzer on the ilastik output (Figure 10, green). Our results demonstrate that while the two analysis pipelines produce equivalent results, the final output is highly dependent on the initial binarization step. It is also important to note that, to ensure a fair comparison, we filtered the MitoLandscape results to exclude objects with a volume less than 0.05 μm^3^, a hardcoded threshold present in the Mitochondria Analyzer plugin. This confirms that a robust and reproducible segmentation strategy, like the one provided by our machine learning model, is crucial for obtaining reliable quantitative data.

Given that segmentation quality emerged as the dominant source of variance in our benchmarks, the following practical guidance on acquisition and pre-processing to maximize mask fidelity should be applied. Use Airyscan for high-SNR super-resolution and enforce Nyquist sampling per channel, followed by Huygens deconvolution. Under our conditions, this yields 120–140 nm XY/350–400 nm Z at 555 nm. This stabilizes segmentations and improves the reliability of downstream MitoLandscape readouts.

Thanks to its modular design, MitoLandscape further allows for the extraction of various spatial metrics, such as distances from soma, nucleus or other organelles, mitochondrial distribution in defined compartments, and volumetric profiles along cellular processes. These types of measurements are especially relevant when working with polarized or highly compartmentalized cells like Radial Glia Cells or neurons. Our analysis is based on geodesic distances and reference points defined by the user, which makes the tool adaptable to different biological scenarios. For example, we automated the classification of processes within the RGCs as apical or basal by integrating information relative to the position of the process with respect to selected tissue landmarks, such as the ventricular surface. This approach can be easily extended to other contexts by incorporating the preferred spatial reference, such as vasculature or pathological structures like amyloid plaques, allowing for organelle distribution studies in a wider range of physiological and disease-related scenarios.

It is important to note that the graph-based modules of MitoLandscape are applied consistently across all sample types, regardless of whether the input is an isolated cultured cell or a cell embedded in dense tissue. What differs is the segmentation step: for isolated cells such as HEK, segmentation is straightforward, whereas in tissue this step becomes critical to separate individual cells with overlapping or intertwined processes. Once segmentation is achieved, the downstream analysis is uniform across all contexts.

The analysis pipeline of MitoLandscape enabled both the quantification of mitochondrial localization and distribution, and the identification of cell-to-cell heterogeneity within the same tissue context. For example, two neighboring radial glial cells displayed distinct mitochondrial density profiles across equivalent compartments, with one cell showing preferential accumulation in the basal process and the other exhibiting enrichment in the apical soma (Figure 8E). The volumetric profiles along apical and basal processes further revealed local hotspots of mitochondrial density, highlighting regions of potential metabolic specialization (Figures 8C,D). In addition, our approach quantified the length of individual mitochondrial elements within each compartment (Figure 8F), and the complexity of mitochondrial elements, quantified as number of branches (Figure 8G). These results illustrate how the pipeline can dissect not only subcellular distribution but also morphological diversity of mitochondria at single-cell resolution, thereby uncovering biologically relevant variability that would be masked by population-level analyses.

While in this study we focused on mitochondria, MitoLandscape may be used to analyze other tubular organelles or cellular structures, if suitable fluorescent reporters are available. Nevertheless, this will require organelle-specific validation given their distinct 3D architecture and spatial distributions. Since the core steps (semi-automated segmentation, pixel classification, and morphological analysis by skeletonization) do not depend on the structure of the specific signal, the method is adaptable to other subcellular structures. In addition, MitoLandscape is ideal to analyze the morphology of neuronal and glial cells, highlighting its versatility in quantifying structures at different biological scales. While originally developed to analyze subcellular components (such as mitochondria), here we show that MitoLandscape is equally useful to capture the detailed architecture of whole cells. Accordingly, this single, unified pipeline is ideally suitable to study both the internal organization of cells and their overall morphology. Indeed, the segmentation and analysis modules can be applied to any biological sample if individual cells and organelles are appropriately labeled, including adult tissues, organoids, or disease models with altered cellular architecture.

In summary, MitoLandscape offers a robust and adaptable tool for the spatial analysis of organelle morphology, architecture and localization within the cell. It enables reproducible, user-friendly and high-throughput quantification of subcellular structures in complex biological samples. We hope that this tool opens the door to more systematic investigations into how organelle organization contributes to cellular identity, development, and function.

## Data Availability

Example pictures used in this study can be found on Zenodo (https://zenodo.org/records/16037033).
