# Supplementary material for: MitoLandscape, a semi-automated pipeline for subcellular localization and quantification of mitochondria
Source: Front Cell Dev Biol. 2025 Nov 7;13:1668779. doi: 10.3389/fcell.2025.1668779 (PMC12635438; doi:10.3389/fcell.2025.1668779)
Supplement: Supplementary file 2 [file DataSheet1.pdf]

Supplementary Material

MitoLandscape, a semi-automated pipeline for subcellular localization and quantification of mitochondria

Enrico Negri, Virginia Fernández, Víctor Borrell

1 Supplementary Figures

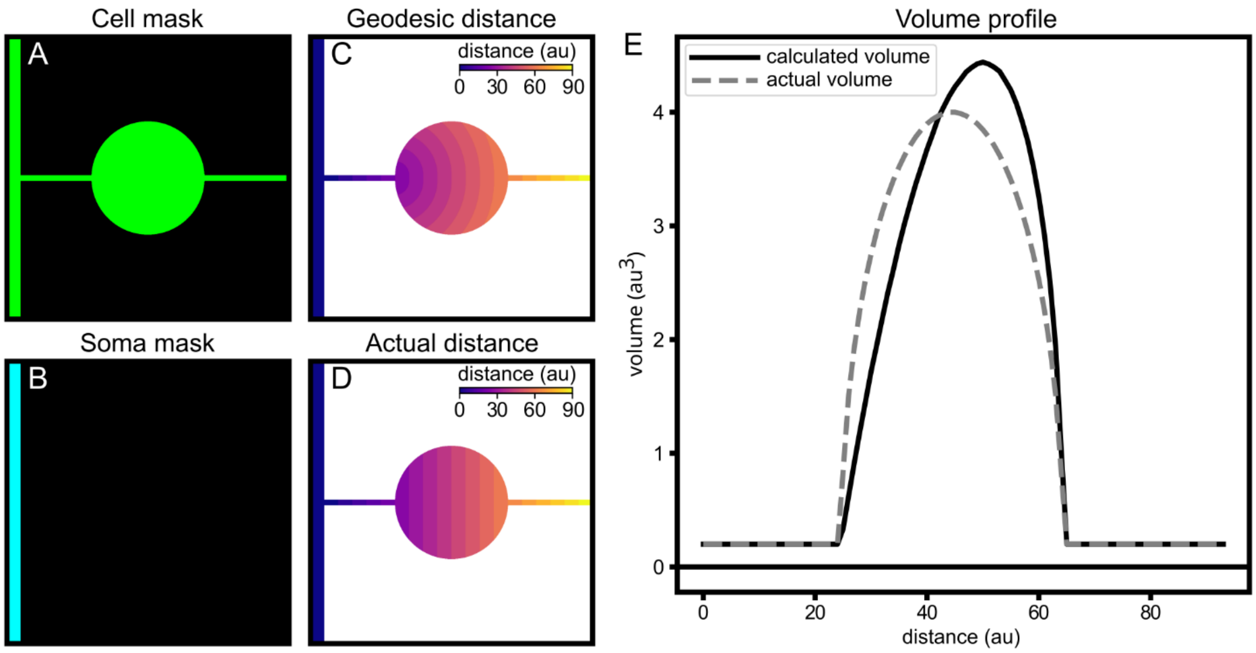

Supplementary Figure 1. Volume profile deformation caused by geodesic distance. (A-D) Theoretical cell (A) and soma (B) masks with a circular varicosity along the process. Volume calculation using geodesic distance from the soma causes a deformation in the slices quantified by the algorithm (C), and actual distance measured in Euclidean distance based on slices perpendicular to the process axis (D). The latter is more accurate because the process is not bending. (E) Plot of the result volume profile comparing the calculated volume (using geodesic distance, solid line) versus the actual volume (using Euclidean distance, dashed line). The thicker the varicosity is compared to the rest of the process, the stronger the deformation.

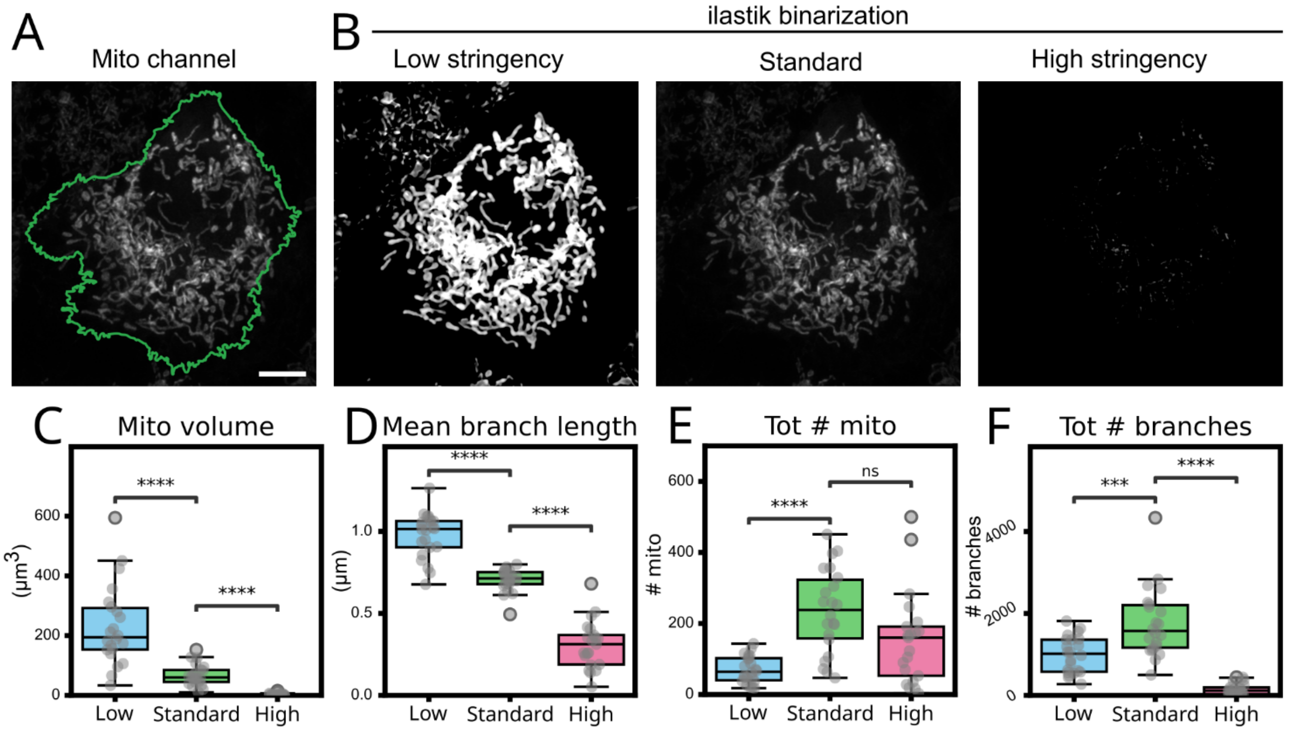

Supplementary Figure 2. Impact of binarization stringency on mitochondrial on quantification in HEK cells. (A) Max projection of mitochondrial channel from a representative HEK cell; in green cell segmentation outline. (B) Average projection of binarized output from three independent two-class ilastik pixel-classification models. Left to right: low stringency (overestimation/false positives), standard (model used for all the HEK analyses), and high stringency (underestimation/false negatives). (C-F) downstream comparisons across models for: (C) total mitochondrial volume per cell, (D) mean branch length, (E) number of mitochondria per cell, and (F) total number of branches per cell. T-test with Bonferroni correction. *: p-value < 0.05, **: p-values < 0.01, ***: p-value < 0.001, ****: p-value < 0.0001. Scale bar: 5 μm.
